# The interaction of behavioral context and motivational-volitional factors for exercise and sport in adolescence: patterns matter

**DOI:** 10.1186/s12889-020-08617-5

**Published:** 2020-04-28

**Authors:** Vanessa Gut, Julia Schmid, Achim Conzelmann

**Affiliations:** Institute of Sport Science, University of Bern, Bremgartenstrasse 145, 3012 Bern, Switzerland

**Keywords:** Latent profile analysis, Social-ecological framework, Organizational and social setting, Psychological factors, Person-oriented approach, Physical activity

## Abstract

**Background:**

In order to generate more effective interventions to promote exercise and sport in adolescence, a better understanding of the interaction of influencing factors across different levels is needed. In particular, motivation and volition for exercise and sport, as well as the context in which adolescents are doing exercise and sport, have been identified as important factors. Behavioral context refers to both the organizational setting, e.g., doing exercise and sport in a club, and the social setting, e.g., doing exercise and sport with friends. Extending previous research, the present study applies a person-oriented approach and aims to identify typical behavioral context patterns and motivational-volitional patterns. To validate the patterns, it was examined whether they differ concerning the exercise and sport activity level. Furthermore, the study investigated how behavioral context patterns and motivational-volitional patterns interact.

**Method:**

A cross-sectional design with 1155 adolescents (*M*_age_ = 15.29; 53% female) was applied. A latent profile analysis was used twice to identify typical patterns: once with eight organizational and social setting factors to examine behavioral context patterns, and once with five motivational-volitional factors to examine motivational-volitional patterns. To validate the patterns identified, the exercise and sport activity level were compared across the patterns using Wald-tests. Finally, transition probabilities and odds ratios were calculated in order to investigate the interaction of the behavioral context and motivational-volitional patterns.

**Results:**

Four behavioral context patterns − differing in activity level − were identified: Mostly inactive, non-club-organized individualists, self-organized individualists and family sportspersons, and traditional competitive club athletes with friends. Furthermore, five motivational-volitional patterns emerged with differing activity levels: three level patterns with overall low, moderate or high motivation and volition, and two shape patterns called the intention- and plan-less and the plan-less motivated. Regarding interaction, the results indicate that one behavioral context pattern is not solely responsible for moderate to high motivation and volition in adolescents.

**Conclusion:**

Applying a person-oriented approach allows a more differentiated view of how behavioral context and motivational-volitional factors interact within homogenous subgroups. This, in turn, provides a basis to design tailored multilevel interventions which account for the interaction of influencing factors across different levels.

## Background

Adolescents as a target group for exercise and sport promotion are interesting for two major reasons: the sharp reduction of exercise and sport activities in this age group [1] and the link between adolescent and lifelong maintenance of exercise and sport behavior [2]. Furthermore, exercise and sport have various positive effects on the biopsychosocial health of individuals [3, 4].

Most of the interventions for promoting exercise and sport show little effect on behavior [5, 6]. To improve their effectiveness, further research is indicated. Firstly, we need to know which factors influence exercise and sport behavior and how they interact with each other [7, 8]. Secondly, we need to figure out which interventions suit which people. As no intervention works equally for everyone, a more differential perspective in exercise and sport promotion is necessary [9, 10]. One strategy is to identify homogenous subgroups among adolescents and to tailor programs to them respectively [11].

According to the social-ecological framework [12], the behavioral context influences exercise and sport in adolescents. The behavioral context refers among others to the organizational setting. So far, empirical research has focused on either club-organized, non-club-organized or self-organized activities [13, 14]. A club-organized setting is typically characterized by regular training sessions and the expectation that club members will voluntarily help out with additional club activities. Non-club-organized settings (e.g., commercial providers, such as gyms) have a similar organizational structure, but fewer social obligations [15]. Furthermore, self-organized, informal settings are more flexible and often have also few obligations. In addition, adolescents can engage in competitive, or more recreational, non-competitive settings [14, 16, 17].

However, the behavioral context not only refers to the organizational, but also to the social setting of exercise and sport [8, 18]. Adolescents can be active together with family members, friends, and people they do not really know as well as doing exercise and sport alone [13, 19]. Previous research [8, 18, 20] has identified underlying mechanisms, such as role modelling as well as emotional and instrumental support that promote adolescents’ exercise and sport behavior.

In addition to the behavioral context, research has identified intrapersonal factors, such as motivation and volition, as central variables for exercise and sport behavior [8, 21]. According to the Self-Determination Theory [22], self-determined motivation is an important factor for adopting and maintaining exercise and sport [7, 23], ranging from self-determined motivation, where a person pursues an activity because the incentive is inherent in the activity itself, to non-self-determined motivation, where a person pursues an activity due to external reasons, such as external pressure or reward [24]. Based on the Health Action Process Approach (HAPA) [25], intention is another central influencing factor for exercise and sport behavior in adolescence [8]. In contrast to the qualitative aspect of self-determined motivation, intention illustrates a quantitative aspect of motivation. It is defined as an individuals‘s decision to perform a behavior with a certain intensity [26]. Besides motivational processes, volitional processes are deemed necessary to transform intention into concrete action [25]. Maintenance self-efficacy has been declared theoretically and empirically important to implement certain behavioral intention [8, 27, 28] and may be defined as the ability to maintain exercise and sport behavior in the long term [29]. Furthermore, action planning has recently received more scientific attention [25, 27, 28]. This involves the precise planning of an activity in terms of when, where, how and with whom the person will initiate a specific behavior [30].

However, in order to foster adolescents’ exercise and sport behavior, it is important to more deeply understand the interplay of influencing factors from different levels [7, 10, 12]. Multilevel studies regarding specific interacting mechanisms are of practical use as they generate knowledge for designing more effective interventions [12, 31]. In fact, a basic tenet of the social-ecological framework [12] is that factors from multiple levels, such as behavioral context and intrapersonal factors, interact with each other. There is evidence that participating in organized activities [17, 32] and in competitions [33], or doing activities with friends [34, 35] all foster motivation and/or volition for exercise and sport. However, most current research has focused on the average interaction effect of behavioral context and intrapersonal factors across a whole population. It is often disregarded that there might be subgroups of adolescents with distinct configurations of variables and interaction mechanisms [9]. For example, it can be assumed that for some adolescents an informal, non-competitive setting could be more motivating than a traditional competitive club-organized setting [17].

To investigate both interactions within and differences between individuals, the person-oriented approach seems theoretically and methodologically appropriate [36–38]. It is rooted in the holistic-interactionistic paradigm [36] where it is assumed that there are interactions between the person (e.g., motivational-volitional factors) and the environment (e.g., organizational and social setting), as well as within both the person and the environment: an individual‘s various factors do not develop and function independently of one another, but rather in a complex, reciprocal interplay. The person-oriented approach focuses on patterns, which means that configurations of variable values within a person are analyzed. As an additional tenet, the person-oriented approach aims to identify homogenous subgroups with typical patterns. This procedure is in contrast to the variable-oriented approach, in which the average effect of one or more variables across a whole population is investigated [38].

Although person-oriented research has grown in recent years [39], the majority of these studies has had a relatively narrow focus. For example within the organizational setting, the research group around Borgers and Scheerder [16, 40] found different organizational patterns in young adults, such as a traditional-competitive pattern and a more recreational, informal pattern. Furthermore, Lawler and colleagues [41] examined the relationship between different behavioral context patterns, such as organized and non-organized sport, and intrinsic motivation among adolescents. Within the social setting, Smith and colleagues [42] found that patterns with more positive peer relationships are associated with higher intrinsic motivation for exercise and sport. However, to our knowledge, there is still little investigation into patterns with a combination of organizational and social setting variables. Up to now, a growing body of research has applied a person-oriented approach to investigate motivation, though this is limited to modes of self-determined motivation [43–47]. Very few person-oriented studies have broadened the area of constructs (e.g., intention) [48] and examined the association with exercise and sport behavior [47].

There is a need to broaden the focus to not only consider patterns of both behavioral context factors and motivational-volitional factors, but also to look at interactions across these two levels. Therefore, this exploratory study aims to investigate the interplay between behavioral context and motivational-volitional factors, using a person-oriented approach. In light of this, the following three research questions were posed:

1. Which behavioral context patterns in exercise and sport in adolescents can be identified?
  To answer this question, four organizational setting factors − club-organized, non-club-organized, self-organized setting and participation in competition − as well as four social setting factors − doing exercise and sport with friends, family/partner, people you do not know, or alone − were included in the study. So far, the few person-oriented studies conducted have focused on only one of these two behavioral contexts. To validate the patterns identified, differences in exercise and sport activity level were investigated. Previous variable-oriented research [7, 8] indicates that adolescents doing exercise and sport in a competitive club-organized setting and with friends tend to have a higher activity level.
2. Which motivational-volitional patterns in exercise and sport in adolescents can be identified?
  Therefore, three motivational factors - self-determined motivation, non-self-determined motivation and intention - as well as two volitional factors - maintenance self-efficacy and action planning - were investigated in this study. Recent person-oriented studies in the motivational-volitional area were very close theoretically, whereby the motivational-volitional area, with its many variables, has not yet been fully covered. To validate the patterns found, it was investigated whether these patterns differ in their exercise and sport activity level. Current variable-oriented research [25, 27] indicates that both motivational and volitional factors are necessary to be physically active. Therefore, it is hypothesized that the higher the motivation and volition in the patterns, the higher the exercise and sport activity level. However, it can also be speculated that the lack of a motivational or volitional factor might be compensated by another factor.
3. How do the behavioral context patterns interact with the motivational-volitional patterns for exercise and sport in adolescents?
  Because of the negligible amount of current research on the interaction of behavioral context patterns and motivational-volitional patterns, there is still little knowledge about this relationship.

## Method

### Design and participants

We applied a cross-sectional research design. The sample consists of 1155 9th grade students (*M*_age_ = 15.29, *SD*_age_ = 0.65; 53% female and 47% male) from 79 classes in lower secondary and baccalaureate schools in the German-speaking part of Switzerland. Of all students, 19% are foreign nationals. Furthermore, 15% attend a class with basic scholastic requirements, 84% a class with extended scholastic requirements and 1% a class without consideration to level differences.

### Procedure

Students filled out a paper-pencil-version of the questionnaire during class hours under the supervision of a trained research team member in spring, 2016. All participants gave their written informed consent and were free to decline participation. Additionally, adolescents under 16 required written confirmation from their parents to participate. The Ethics Committee of the University of Bern‘s Faculty of Human Sciences approved the study.

### Measures

#### Exercise and sport activity level and behavioral context

We used a well-validated German questionnaire by Fuchs et al. [49] to assess exercise and sport behavior. The participants were asked to note their activities and to indicate how many times in the last 4 weeks and for how many minutes each time, they engaged in this exercise and sport activity. Furthermore, adolescents were asked about the behavioral context of their activities. (1) They were asked in which organizational setting they undertook exercise and sport: (a) club-organized, (b) non-club-organized, or (c) self-organized, and (d) in which social setting they did the exercise and sport activity: (a) with friends, (b) with family and/or partner, (c) with a person they do not know very well, or (d) alone.

#### Self-determined motivation

We assessed self-determined motivation by using the sport-and exercise-related self-concordance-scale [50]. The validated German scale includes 12 items with four subscales, each with three items: intrinsic (α = .75), identified (α = .73), introjected (α = .80), and external modus (α = .70) of motivation for exercise and sport [50]. Participants ranked their motivation on a Likert-scale from 1 (*I strongly disagree*) to 6 (*I strongly agree*). An index of self-determined motivation (mean value of intrinsic and identified modus) and an index of non-self-determined motivation (mean value of introjected and external modus) were then calculated.

#### Intention strength

To assess the strength of the intention to exercise and take part in sport, we used a German scale by Seelig and Fuchs [50]. The adolescents were asked how strong their intention was to regularly do exercise and sport in the next weeks and months in their leisure time. The adolescents stated their answer using a Likert-scale from 1 (*not at all*) to 10 (*very strong*).

#### Maintenance self-efficacy

To assess self-efficacy, we used a 10-item-scale by Sniehotta et al. [51], which was specifically adapted for German-speaking adolescents [52] and received good internal consistency (α = .83). The adolescents were asked, for example, how sure they are that they will undertake regular exercise and sport, even though they have a lot to do. They stated their answers on a Likert-scale from 1 (*not at all)* to 5 (*absolutely*).

#### Action planning

We measured action planning with a five-item-scale by Sniehotta et al. [51], specifically adapted for German-speaking adolescents [52], and received good internal consistency (α = .87). Adolescents were asked how precisely they planned their exercise and sport activities in the next weeks (e.g., “I have already planned precisely when I will be doing exercise and sport”). They stated their answers on a Likert-scale from 1 (*not at all)* to 5 (*absolutely*).

### Data preparation and analysis

Four individuals were excluded due to missing values in all relevant variables. We controlled the sample for multivariate outliers (Mahalanobis distance values as *χ*^2^ at *p* < .001 [53];) and, thus, excluded five individuals. Furthermore, the activity level was used to convert the information of organizational and social setting into percentage variables. Missing values were estimated by means of the default full information maximum likelihood procedure [54]. Missing values were all < 5%.

We used latent profile analysis (LPA) [55–57] with correction for nested data in terms of school classes to identify behavioral context patterns and motivational-volitional patterns. LPA aims to classify individuals with similar patterns to the same latent subgroup considering its probability of class membership [39]. In a first step, we conducted a series of one to eight LPA-models with Mplus Version 8 [58] to separately identify behavioral context as well as motivational-volitional patterns. To identify behavioral context patterns, eight context variables were included in the LPA (see Table 1). To investigate motivational-volitional patterns, we used five variables (see Table 1). We took a combination of statistical and theoretical indicators into consideration to identify the optimal number of patterns. As statistical indicators, we used the log likelihood value, Bayesian information criterion (BIC), entropy, Vuong-Lo-Mendell-Rubin likelihood ratio test (VLMR) and bootstrap likelihood ratio test (BLRT) [55, 56, 59]. Morin and Wang [57] recommend using the statistical indicators to form graphical elbow-plots. However, in the end, theoretical indicators, such as the principle of parsimony, theoretical considerations and the interpretability of the identified patterns, were decisive in choosing the optimal number of patterns [59]. In addition, random split-half replication [60] of the patterns was used as a further criterion. To validate the patterns, we used Wald-tests to investigate if the identified patterns differed in terms of exercise and sport activity level [61]. In a final step, we calculated transition probabilities and odds ratios (OR) to examine the interaction of behavioral context patterns and motivational-volitional patterns.

**Table 1:** Descriptive statistics and correlations of behavioral context and motivational-volitional factors, as well as exercise and sport activity level

Variables	*M*	*SD*	α	1	2	3	4	5	6	7	8	
Organizational and social setting factors												
1. Club-organized	40.60%	43.50	–	–								
2. Non-club-organized	13.20%	29.80	–	−.32*	–							
3. Self-organized	24.50%	36.50	–	−.41*	−.19*	–						
4. Alone	16.50%	43.90	–	.49*	.13*	.42*	–					
5. With people you do not know	8.40%	17.00	–	−.21*	.11*	−.07*	−.11*	–				
6. With family and/or partner	5.00%	23.50	–	.13*	.06*	.20*	−.09*	−.04	–			
7. With friends	51.60%	29.80	–	−.09*	.00	−.07*	−.39*	−.28*	−.19*	–		
8. Competition participation	34.50%	42.80	–	.69*	−.19*	−.24*	−.16*	−.00	−.04	.47*	–	
Exercise and sport activity level (min/week)	237.21	242.20	–	.29*	.05	.11*	.07*	−.02	.05	.34*	.39*	
Motivational-volitional factors	*M*	*SD*	α	1	1a	1b	2	2a	2b	3	4	5
1. Self-determined motivation index (ranging from 1 to 5)	4.35	1.07	–									
a) Intrinsic modus of motivation	4.16	1.29	.75	.89*								
b) Identified modus of motivation	4.53	1.15	.73	.87*	.55*							
2. Non-self-determined motivation index (ranging from 1 to 5)	2.33	0.99	–	.25*	.12*	.33*						
a) External modus of motivation	1.80	0.93	.70	.11*	.07	.12*	.77*					
b) Introjected modus of motivation	2.87	1.39	.80	.28*	.12*	.39*	.91*	.43*				
3. Intention strength (ranging from 1 to 10)	8.02	2.03	–	.64*	.62*	.49*	.07	−.04	.13*			
4. Maintenance self-efficacy (ranging from 1 to 5)	3.31	0.79	.83	.60*	.58*	.46*	.14*	.04	.18*	.52*		
5. Action planning (ranging from 1 to 5)	3.53	1.10	.87	.57*	.54*	.45*	.14*	.04	.17*	.63*	.53*	
Exercise and sport activity level (min/week)	237.21	242.20	–	.40*	.43*	.26*	.08*	.04	.09*	.41*	.38*	.38*

*p < .05

## Results

### Behavioral context patterns

The descriptive statistics and correlations between all the variables are available in Table 1. We addressed research question 1 by running LPA. BLRTs were not significant (*p* < .05; see Table 2). Based on VLMR, a five-pattern-solution can be assumed. The elbow-criterion for log likelihood and BIC pointed to a three- to-five pattern-solution (see Table 2 and electronical supplement material [ESM] 1). The three-pattern-solution indicated too little differentiation, because the patterns were mainly characterized by the organizational setting, such as club-organized, non-club-organized or self-organized. On the other hand, in the five-pattern-solution, one parameter has to be fixed, indicating that the model is too complex. Thus, we favored a four-pattern-solution based on content-related criteria, such as theoretical considerations and the principle of parsimony. Furthermore, the decision for the four-pattern-solution was reinforced by the fact that the solution was replicated with two random split-half samples (see ESM 2). Pattern one was labelled as *mostly inactive* (*n* = 240, 20.94%), whereby the majority of adolescents (82.90%, see ESM 3) were completely inactive. Pattern two was labelled as *non-club-organized individualists* (*n* = 150, 13.18%). In this pattern, adolescents often do activities alone in a non-club-organized setting. For example, they do individual sports, such as dancing or different fitness workouts in gyms. The adolescents in pattern three were called *self-organized individualists and family sportspersons* (*n* = 254, 22.16%) because of their characteristically high percentage of exercise and sport activities undertaken alone and with family members in a self-organized setting. They were doing a broad variety of exercise and sport activities, such as jogging or playing football. In the last pattern, adolescents were called *traditional competitive club athletes with friends* (*n* = 501, 43.72%), whereby the adolescents are characterized by a high percentage of doing exercise and sport in a club with their friends in a competitive setting. Adolescents in this pattern often do team sports, such as football, handball or floorball. For a more detailed insight into further descriptive characteristics of the patterns, see ESM 3. Regarding exercise and sport activity level (See Table 2), the identified patterns differ significantly (χ^2^ = 921.71, *p* < .00005). The mostly inactive have the lowest activity level, at 17.87 min per week, whereby the traditional competitive club athletes with friends enjoy the highest activity level, at 324.42 min per week. The activity level of the two other patterns, non-club-organized individualists (261.82 min/week) and self-organized individualists and family sportspersons (262.02 min/week), do not differ significantly.

**Table 2:** Behavioral context patterns: latent profile analysis models for 3- to 5-latent-patterns-solutions (class-invariant, diagonal Σ) including correction for nesting

Latent-patterns-solution	*n* (%)	Club-organized *M (SD)*	Non-club organized *M (SD)*	Self-organized *M (SD)*	Alone *M (SD)*	With people you do not know *M (SD)*	With family and/or partner *M (SD)*	With friends *M (SD)*	Competition participation *M (SD)*	Exercise and sport activity level *M (SD)*	BIC	Entropy	VLMR	BLRT^a^
*Three latent patterns*											1175.63	.98	*p* < .00005	− 1414.251 *p* < .00005
1. Self-organized sportpersons	493 (43.02%)	4.10% (14.49)	1.40% (1.00)	45.70% (32.09)	19.80% (30.66)	3.80% (23.24)	6.00% (17.32)	27.80% (37.82)	7.10% (37.82)	139.94 min/week (9.74) [2, 3]				
2. Non-club-organized sportpersons	150 (13.09%)	3.60% (14.49)	86.10% (1.00)	9.40% (32.09)	29.50% (30.66)	13.50% (23.24)	7.50% (17.32)	48.70% (37.82)	12.20% (37.82)	263.41 min/week (19.38) [1, 3]				
3. Traditional competitive club athletes with friends	503 (43.89%)	87.90% (14.49)	2.90% (1.00)	8.00% (32.09)	9.30% (30.66)	11.50% (23.24)	3.10% (17.32)	76.10% (37.82)	68.40% (37.82)	324.81 min/week (10.67) [1, 2]				
									Wald-Chi-Square-Test	χ^2^ = 164.08, *p* < .00005				
*Four latent patterns*											− 389.82	.99	*p* < .00005	− 457.082 *p* < .00005
1. Mostly inactive	240 (20.94%)	0.50% (14.14)	0.60% (0.10)	1.00% (13.78)	0.80% (28.11)	1.70% (23.02)	0.50% (17.03)	9.40% (35.92)	1.40% (31.31)	17.87 min/week (4.75) [2–4]				
2. Non-club-organized individualists	151 (13.18%)	3.70% (14.14)	85.90% (0.10)	9.50% (13.78)	29.50% (28.11)	13.80% (23.02)	7.60% (17.03)	48.70% (35.92)	12.20% (31.31)	261.81 min/week (19.30) [1, 4]				
3. Self-organized individualists and family sportpersons	254 (22.16%)	7.50% (14.14)	1.80% (0.10)	88.70% (13.78)	38.20% (28.11)	5.40% (23.02)	11.10% (17.03)	45.00% (35.92)	13.40% (31.31)	262.02 min/week (15.57) [1, 4]				
4. Traditional competitive club athletes with friends	501 (43.72%)	87.80% (14.14)	3.10% (0.10)	7.70% (13.78)	9.00% (28.11)	22.60% (23.02)	3.10% (17.03)	76.20% (35.92)	67.90% (31.31)	324.42 min/week (10.50) [1–3]				
									Wald-Chi-Square-Test	χ ^2^ = 921.71, *p* < .00005				
*Five latent patterns*											− 1617.68	.99	*p* = .0236	365.529 *p* < .00005
1. Mostly inactive	238 (20.77%)	0.00% (7.75)	0.30% (10.00)	0.80% (12.25)	0.70% (27.75)	1.50% (23.02)	0.50% (17.03)	8.50% (35.64)	1.10% (31.15)	14.37 min/week (3.84) [2–5]				
2. Organizationally flexible athletes	168 (14.66%)	54.80% (7.75)	10.10% (10.00)	31.30% (12.25)	18.80% (27.75)	11.20% (23.02)	7.10% (17.03)	63.20% (35.64)	52.50% (31.15)	400.12 min/week (20.19) [1, 3–5]				
3. Traditional competitive club athletes with friends	379 (33.07%)	96.50% (7.75)	0.70% (10.00)	2.40% (12.25)	6.50% (27.75)	11.80% (23.02)	2.30% (17.03)	79.40% (35.64)	70.60% (31.15)	290.31 min/week (11.32) [1, 2, 5]				
4. Non-club-organized sportspersons	146 (12.74%)	2.10% (7.75)	87.30% (10.00)	9.70% (12.25)	29.70% (27.75)	14.20% (23.02)	7.60% (17.03)	47.90% (35.64)	11.50% (31.15)	255.55 min/week (19.57) [1, 2]				
5. Self-organized individualists	215 (18.76%)	1.80% (7.75)	2.00% (10.00)	94.00% (12.25)	40.50% (27.75)	4.30% (23.02)	11.00% (17.03)	43.70% (35.64)	9.40% (31.15)	247.98 min/week (16.53) [1–3]				
									Wald-Chi-Square-Test	χ^2^ = 1087.54, *p* < .00005				

*BIC* Bayesian information criterion, *VLMR* Vuong-Lo-Mendell-Rubin likelihood ratio test, *BLRT* bootstrapped likelihood-ratio test. Numbers in brackets indicate that patterns differ significantly at *p* < .05

^a^The BLRT could only be performed without correction for nesting

### Motivational-volitional patterns

To identify motivational-volitional patterns, one- to eight-pattern-solutions were compared. The elbow-criterion for log likelihood and BIC pointed to a four- to six-pattern-solution (see Table 3 and ESM 1). A deeper inspection of the patterns (see Table 3) showed that the four-pattern-solution is not sufficiently differentiated in terms of content. Based on theoretical considerations, both the five- and six-pattern-solution are meaningful. However, the VLMR pointed to the five-pattern-solution (*p* > .05). Therefore, we favored a five-pattern-solution, which was also replicated with two random split-half-samples (ESM 2). The adolescents in pattern one are characterized by very low levels of maintenance self-efficacy and action planning and therefore labelled *the intention- and plan-less* (*n* = 61, 5.32%) (see Table 3 and ESM 3). The adolescents in pattern two have overall low motivation and volition and were, therefore, called *the low motivated with low volition* (*n* = 152, 13.26%). In pattern three, adolescents with overall moderate motivation and volition, were called *the moderately motivated with moderate volition* (*n* = 414, 36.13%). The adolescents in pattern four are characterized by above-average motivation, but low planning and are, therefore, labelled *the plan-less motivated* (*n* = 42, 3.67%). Adolescents in pattern five are characterized by overall high motivation and volition and are called *the highly motivated with high volition* (*n* = 477, 41.62%). For a more detailed insight into further descriptive characteristics of the patterns, see ESM 3. Regarding exercise and sport activity level (see Table 3), the five identified patterns differ significantly (χ^2^ = 577.69, *p* < .00005). The intention- and plan-less (18.19 min/week) have the lowest activity level, followed by the low motivated with low volition (88.45 min/week). The activity levels of the moderately motivated with moderate volition (171.94 min/week) and the plan-less motivated (237.94 min/week) do not differ significantly. The most active adolescents are the highly motivated with high volition, at 327.18 min per week.

**Table 3:** Motivational-volitional patterns: latent profile analysis models for 4- to 6-latent-patterns-solutions (class-invariant, diagonal Σ) including correction for nesting

Latent-patterns-solution	*n* (%)	Self-determined motivation *M (SD)*	Non-self-determined motivation *M (SD)*	Intention strength *M (SD)*	Self-efficacy *M (SD)*	Action planning *M (SD)*	Exercise and sport activity level *M*_min/week_*(SD)*	BIC	Entropy	VLMR	BLRT^a^
*Four latent patterns*								15,663.99	.76	*p* = .0861	*− 7807.680* *p* < .00005
1. The moderately motivated with moderate volition	442 (38.57%)	4.29 (0.70)	2.31 (0.97)	8.21 (1.13)	3.11 (1.13)	3.47 (0.71)	184.13 min/week (11.87) [2–4]				
2. The intention- and plan-less	62 (5.41%)	2.11 (0.70)	1.64 (0.97)	2.73 (1.13)	2.15 (1.13)	1.31 (0.71)	19.04 min/week (8.33) [1, 3, 4]				
3. The above-average motivated with above-average volition	174 (15.18%)	3.29 (0.70)	2.27 (0.97)	6.00 (1.13)	2.63 (1.13)	2.41 (0.71)	89.30 min/week (16.87) [1, 2, 4]				
4. The highly motivated with high volition	468 (40.84%)	5.10 (0.70)	2.47 (0.97)	9.31 (1.13)	3.91 (1.13)	4.31 (0.71)	372.70 min/week (14.18) [1–3]				
						Wald-Chi-Square-Test	χ^2^ = 577.694, *p* < .0005				
*Five latent patterns*								15,591.81	.80	*p* = .0069	− 7733.380 *p* < .00005
1. The intention- and plan-less	61 (5.32%)	2.12 (0.70)	1.63 (0.97)	2.70 (1.14)	2.17 (0.57)	1.25 (0.58)	18.19 min/week (8.48) [2–5]				
2. The low motivated with low volition	152 (13.26%)	3.24 (0.70)	2.27 (0.97)	5.90 (1.14)	2.59 (0.57)	2.33 (0.58)	88.45 min/week (16.37) [1, 3–5]				
3. The moderately motivated with moderate volition	414 (36.13%)	4.23 (0.70)	2.35 (0.97)	8.05 (1.14)	3.07 (0.57)	3.59 (0.58)	171.94 min/week (11.64) [1, 2, 5]				
4. The plan-less motivated	42 (3.67%)	4.59 (0.70)	2.05 (0.97)	8.72 (1.14)	3.33 (0.57)	1.82 (0.58)	237.94 min/week (47.43) [1, 2, 5]				
5. The highly motivated with high volition	477 (41.62%)	5.08 (0.70)	2.46 (0.97)	9.31 (1.14)	3.89 (0.57)	4.36 (0.58)	372.18 min/week (13.83) [1–4]				
						Wald-Chi-Square-Test	χ^2^ = 577.69, *p* < .0005				
*Six latent patterns*								15,572.57	.81	*p* = .6366	− 7676.156 *p* < .00005
1. The moderately motivated with moderate volition	393 (34.29%)	4.25 (0.72)	2.31 (0.97)	8.23 (0.99)	3.07 (0.57)	3.57 (0.57)	177.98 min/week (11.79) [2, 4, 5]				
2. The intention- and plan-less	58 (5.06%)	2.19 (0.72)	1.68 (0.97)	2.56 (0.99)	2.21 (0.57)	1.27 (0.57)	20.87 min/week (8.28) [1, 3–6]				
3. The external motivated with high planning	50 (4.36%)	4.02 (0.72)	2.76 (0.97)	5.33 (0.99)	3.10 (0.57)	3.43 (0.57)	116.76 min/week (37.43) [1, 2, 5, 6]				
4. The low motivated with low volition	138 (12.04%)	3.16 (0.72)	2.21 (0.97)	6.08 (0.99)	2.53 (0.57)	2.16 (0.57)	87.74 min/week (18.75) [1, 2, 5, 6]				
5. The highly motivated with high volition	470 (41.01%)	5.08 (0.72)	2.46 (0.97)	9.36 (0.99)	3.90 (0.57)	4.37 (0.57)	372.32 min/week (13.94) [1–3]				
6. The plan-less motivated	37 (3.23%)	4.59 (0.72)	2.02 (0.97)	8.93 (0.99)	3.37 (0.57)	1.79 (0.57)	250.33 min/week (50.04) [2–5]				
						Wald-Chi-Square-Test	χ^2^ = 566.34, *p* < .0005				

*BIC* Bayesian information criterion, *VLMR* Vuong-Lo-Mendell-Rubin likelihood ratio test, *BLRT* bootstrapped likelihood-ratio test. Numbers in brackets indicate that patterns differ significantly at *p* < .05

^a^The BLRT could only be performed without correction for nesting

### Association of behavioral context patterns and motivational-volitional patterns

In order to investigate the association of behavioral context and motivational-volitional patterns, the two pattern-solutions were analyzed (see Table 4 or for a graphical representation, see ESM 4). On a descriptive level, 42.30% of the mostly inactive were characterized as the low motivated with low volition. Within the non-club-organized individualists, 43.60% of the adolescents belong to the moderately motivated with moderate volition and 46.20% to the highly motivated with high volition. In the pattern of the self-organized individualists and family sportspersons 44.60% belong to the moderately motivated with moderate volition. The majority (61.90%) of the traditional competitive club athletes with friends are characterized by the highly motivated with high volition. Considering OR (see Table 4 and ESM 4), adolescents classified as non-club-organized individualists have a higher chance of belonging to the moderately motivated with moderate volition (OR = 49.70) and to the highly motivated with high volition (OR = 377.28). Furthermore, adolescents categorized as self-organized individualists and family sportspersons have an increased chance of belonging to the following three patterns: the moderately motivated with moderate volition (OR = 11.62), the plan-less motivated (OR = 16.12) and the highly motivated with high volition (OR = 55.31). A similar result has been shown by the traditional competitive club athletes with friends. Adolescents in this pattern have a higher chance of belonging to the moderately motivated with moderate volition (OR = 25.66), to the plan-less motivated (OR = 11.86) and to the highly motivated with high volition (OR = 353.19). Overall, the results indicate that one behavioral context pattern is not solely responsible for moderate to high motivation and/or volition in adolescents.

**Table 4:** The association of behavioral context patterns with motivational-volitional patterns expressed as transition probabilities and odds ratios

	Motivational-volitional patterns (*N* = 1146)														
	1. The intention- and plan-less (*n* = 61; 5.32%)			2. The low motivated with low volition (*n* = 152; 13.26%)			3. The moderately motivated with moderate volition (*n* = 414; 36.13%)			4. The plan-less motivated (*n* = 42; 3.67%)			5. The highly motivated with high volition (*n* = 477; 41.62%)		
Behavioral context patterns (*N* = 1146)	Probabilities	OR [95% CI]	Distribution within pattern	Probabilities	OR [95% CI]	Distribution within pattern	Probabilities	OR [95% CI]	Distribution within pattern	Probabilities	OR [95% CI]	Distribution within pattern	Probabilities	OR [95% CI]	Distribution within pattern
1. Mostly inactive *(n* = 240; 20.94%)	20.00%	1 [1; 1]	78.70% (*n* = 48)	42.30%	1 [1; 1]	54.60% (*n* = 83)	29.30%	1 [1; 1]	18.40% (*n* = 76)	4.40%	1 [1; 1]	26.20% (*n* = 11)	4.10%	1 [1; 1]	4.60% (*n* = 22)
2. Non-club-organizedindividualists (*n* = 151; 13.18%)	0.60%	1 [1; 1]	1.60% (*n* = 1)	8.10%	6.40 [0.55; 74.51]	7.90% (*n* = 12)	43.60%	49.70 [4.47; 526.28]*	14.70% (*n* = 61)	1.50%	11.19 [0.54; 233.00]	9.50% (*n* = 4)	46.20%	377.28 [30.76; 4627.81]*	15.30% (*n* = 73)
3. Self-organized individualists and family sportspersons (*n* = 254; 22.16%)	2.60%	1 [1; 1]	11.50% (*n* = 7)	14.10%	2.54 [0.92; 7.05]	25.70% (*n* = 39)	44.60%	11.62 [4.47; 30.19]*	26.10% (*n* = 108)	9.20%	16.12 [4.47; 58.08]*	38.10% (*n* = 16)	29.60%	55.31 [15.32; 199.70]*	17.60% (*n* = 84)
4. Traditional competitive club athletes with friends (*n* = 501; 43.72%)	0.90%	1 [1; 1]	8.20% (*n* = 5)	2.70%	1.51 [0.37; 6.15]	11.80% (*n* = 18)	32.30%	25.66 [8.07; 81.56]*	40.80% (*n* = 169)	2.20%	11.86 [2.57; 54.80]*	26.20% (*n* = 11)	61.90%	353.19 [84.45; 1477.05]*	62.50% (*n* = 298)

*OR* odds ratios, Reference categories are the mostly inactive for the behavioral context patterns and the intention- and plan-less for the motivational-volitional patterns; OR > 1 indicate a higher probability than expected and OR < 1 indicate a lower probability than expected compared to the reference categories. 95% CI = 95% Confident interval for odds ratios

*p < .05 indicates significant OR

## Discussion

The first aim of the current person-oriented study was to identify behavioral context patterns. Four patterns were identified: (a) mostly inactive, (b) non-club-organized individualists, (c) self-organized individualists and family sportspersons, and (d) traditional competitive club athletes with friends. The largest pattern four, is characterized by activities in a competitive, club-organized setting with friends. This is in accordance with existing research [8, 62] showing that the most popular setting for adolescents is doing activities in clubs and with friends. However, the fact that adolescents in pattern two and three had chosen a more informal and flexible setting for their activities indicates that this is not the case for every adolescent. These results are in line with previous studies [16, 17, 40, 62] which highlighted that a smaller group of individuals engage mainly in a non-competitive, informal, and more flexible setting. This setting could be especially beneficial for female adolescents [14, 63]. In addition, the small sample size of pattern three points out that only for a minority of adolescents are family members still an important social source for doing exercise and sport. The increasing significance of friends and the decreasing significance of parents for exercise and sport have been observed, especially in older adolescents [64–66].

With regard to the exercise and sport activity level, adolescents in the pattern of the traditional competitive club athletes with friends are the most active. An explanation for this high level of activity can be seen in the fact that competitive sports require regular club training sessions [40]. For the promotion of exercise and sport, it therefore seems beneficial for adolescents to pursue activities in a competitive club-organized setting. However, with regard to dropout in sports clubs during the transition from adolescence to adulthood [62], a combination of organized and self-organized activities, resulting in a more flexible context pattern from an organizational point of view [41], might be the most promising pattern in the long term. This pattern was also found in the six-pattern-solution showing the highest activity level, also compared to the traditional competitive club athletes with friends (see Table 2).

In a second step, five distinct motivational-volitional patterns were identified. Extending previous work in this field of research [43], we combined diverse factors to cover the motivational-volitional area more comprehensively. The three largest patterns - two, three and five - are characterized by low, moderate, or even high characteristics across all motivational and volitional factors. The emergence of so-called level effects [67] indicates that for most people, these factors are very closely related. However, the finding of the other two, smaller patterns - one and four - indicates that there is a variation of expression of single factors within a small group of adolescents. The shape effects indicated in patterns one and four [67] illustrate complex interaction mechanisms of motivational-volitional factors within the subgroups. For example, adolescents in pattern four can be classified as intenders [25] since they are motivated, especially in terms of intention, but have a low volition.

With regard to the activity level, the comparison across the patterns showed mainly that the more motivation and volition the adolescents have, the more active they are. These results are in accordance with previous variable-oriented studies, revealing a positive association between motivational-volitional factors and exercise and sport behavior [7, 8]. Of particular interest is pattern four because around 80% of these adolescents are active despite low action planning. This phenomenon is contrary to the theoretical assumption [25] that besides motivational factors, volitional factors, such as action planning, are also necessary to initiate concrete action. This is also known as the intention-behavior-gap [68]. An explanation of why the plan-less motivated still manage to maintain their activities, even though they do not plan, could be that favorable context conditions foster maintenance of these activities and thus, compensate for low planning. This is known as compensatory effect [69].

In a final step, we linked the behavioral context patterns with the motivational-volitional patterns. Overall, results show that the three behavioral context patterns two, three and four are associated with the pattern of high motivation and volition. This indicates that not only one favorable behavioral context pattern is associated with high motivation and volition in adolescents. It can, therefore, be assumed that the specific configuration of contextual variables within the patterns are decisive for a specific subgroup of adolescents. For some adolescents a competitive club-organized setting with friends is favorable in terms of their motivation and volition [33, 34], whereas for other adolescents a self-organized setting with family members or alone boosts their motivation and volition more effectively [70].

Another aspect of interest is the association of behavioral context patterns with the pattern of the plan-less motivated. The high percentage of pattern four (traditional competitive club athletes with friends; 26.1%), and of pattern three (self-organized individualists and family sportspersons; 38.1%) represented in the plan-less motivated might explain why these adolescents are still very active despite their low planning. It might be possible that through regular club training at the same time and place, adolescents no longer need to plan their activities. Specific behavioral context could serve as a cue that triggers adolescents’ exercise and sport behavior [71, 72]. This automatic and unconscious process, in turn, does not necessarily need volitional abilities, such as planning [73]. Based on this assumption, a habit, such as an automaticity of doing exercise and sport might be internalized by these adolescents [73]. Similarly, it can be speculated that adolescents doing exercise and sport with family members might profit from these joint activities, since family members take over the planning. Therefore, adolescents who receive social support from family members in the form of doing joint activities [8, 18] may need fewer planning abilities.

Transferring the findings into practical implications, with consideration given to inter-individual differences, helps to tailor interventions for specific subgroups [9, 10, 74]. For example, for the inactive group of the plan-less motivated, volitional interventions [75] and the building of favorable context conditions might be most effective, whereby for the low motivated with low volition individuals, a combination of motivational and volitional interventions [76] as well as the building of favorable context conditions seem to be the most promising approach.

In summary, the results help to better understand and promote adolescents’ exercise and sport behavior. On the one hand, the findings allow one to specify the relatively general assumptions about the interplay of behavioral context and intrapersonal factors in the social-ecological framework. On the other hand, the study might provide a base for developing interventions that address both behavioral context and intrapersonal factors. Such so-called multilevel interventions could be more effective than interventions focusing only on one of these two levels [7, 31, 77].

### Limitations and future research directions

The following critical issues of the study point to future research directions and must be taken into consideration: Firstly, we applied a cross-sectional design in our study that has two important consequences. A first consequence is that no causal statements can be implied by the association of behavioral context patterns and motivational-volitional patterns or exercise and sport behavior. Reciprocal interaction between behavioral context and motivational-volitional factors can be assumed [37], which means that certain patterns reinforce each other. However, as a first attempt to investigate interactions of contextual and motivational-volitional factors based on a person-oriented approach, this study has the advantage of examining relationships within a larger sample. Future longitudinal studies should investigate causal effects of the patterns and how they are associated with exercise and sport behavior. In addition, this knowledge will help to better integrate motivational-volitional theories within the social-ecological framework and to more precisely formulate hypotheses concerning the underlying interaction mechanisms [12, 78]. A second consequence of the cross-sectional design is that the temporal stability of the patterns cannot be investigated. Current research [16, 79, 80], for example, shows that people have the tendency to shift from more traditional, organized settings, such as traditional competitive athletes with friends, to more self-organized settings of exercise and sport, such as the self-organized individualists and family sportspersons. However, it remains unanswered if this is a general trend across the population or if this is especially high during the transition from adolescence to adulthood, accompanied by a change in daily structure, such as the school-to-school-transition [81, 82].

Secondly, exercise and sport activity level was collected solely by means of retrospective self-reports. Nevertheless, since our focus relies on the interaction of contextual and motivational-volitional patterns, the questionnaire used seems to be an appropriate and economical measurement method. However, future studies that include objective methods, such as accelerometers, are recommended to measure the intensity of exercise and sport behavior more accurately [83]. In addition, research should be driven forward to a better understanding of exercise and sport as a complex behavioral system. Therefore, a broader focus, not only on quantitative aspects, such as the activity level, but also on qualitative aspects, such as the type of activity, e.g., team or individual sports [41, 84] may be useful.

Finally, the application of LPA as a relatively new and exploratory method [57] can be viewed critically. Although LPA has statistical criteria, there is still a degree of subjectivity inherent in the method concerning the decision of the optimal number of patterns. Thus, in future, replication of the patterns found across different samples is necessary. Besides, there is an ongoing methodological debate about the best way to disentangle level from shape effects in LPA [67, 85].

## Conclusions

In conclusion, the results of this study support the application of a person-oriented approach [36, 37] to better explain influencing factors of adolescents’ exercise and sport behavior, shedding a more differentiated light on the interacting effects of behavioral context and motivational-volitional factors within different subgroups. This new approach helps to generate target-group-specific knowledge and this, in turn, provides the basis to plan tailored multilevel programs to more efficiently promote exercise and sport in adolescents [7]. Ultimately, the aim is to enable adolescents to embrace an active lifestyle across their lifespan that leads to a variety of health benefits [3].

## Supplementary information

**Additional file 1 :** ESM 1. Model fit indices for latent profile analysis of behavioral context and motivational-volitional patterns with correction for nesting.

**Additional file 2 :** ESM 2. Split-half-replication of behavioral context and motivational-volitional patterns.

**Additional file 3 :** ESM 3. Descriptive statistics of behavioral context and motivational-volitional patterns.

**Additional file 4 :** ESM 4. Behavioral context patterns and the association with motivational-volitional patterns expressed as odds ratios with 95% confidence intervals.

## Data Availability

The datasets and syntaxes used during the current study are available from the corresponding author on request.

## Abbreviations

BIC: Bayesian information criterion
BLRT: Bootstrap likelihood ratio test
ESM: Electronical supplement material
HAPA: Health Action Process Approach
LPA: Latent profile analysis
OR: Odds ratio
VLMR: Vuong-Lo-Mendell-Rubin likelihood ratio test
