# Supplementary material for: The interaction of behavioral context and motivational-volitional factors for exercise and sport in adolescence: patterns matter
Source: BMC Public Health. 2020 Apr 28;20:570. doi: 10.1186/s12889-020-08617-5 (PMC7189603; doi:10.1186/s12889-020-08617-5)
Supplement: Supplementary file 1 — Additional file 1 : ESM 1. Model fit indices for latent profile analysis of behavioral context and motivational-volitional patterns with correction for nesting. [file 12889_2020_8617_MOESM1_ESM.docx]

ESM 1

Table 1

*Model fit indices for latent profile analysis of behavioral context and motivational-volitional patterns with correction for nesting.*

| Profile | LL | *df* | BIC | Entropy | VLMR | BLRT^1^ |  |
| --- | --- | --- | --- | --- | --- | --- | --- |
| Behavioral context patterns (*n* = 1146) | | | | | | |  |
| 1 | -2662.02 | 16 | 5436.75 | 1.00 | / | / |  |
| 2 | -1411.58 | 25 | 2999.26 | 0.98 | < .00005 | < .00005 |  |
| 3 | -468.067 | 34 | 1175.63 | 0.98 | < .00005 | < .00005 |  |
| 4 | 346.35 | 43 | -389.82 | 0.99 | < .00005 | < .00005 |  |
| 5 | 991.99 | 52 | -1617.68 | 0.99 | .02 | < .00005 |  |
| 6 | 1592.07 | 61 | -2754.44 | 0.99 | .15 | < .00005 |  |
| 7 | 2144.68 | 70 | -3796.28 | 0.98 | .13 | < .00005 |  |
| 8 | 2625.57 | 79 | -4694.66 | 0.99 | .00 | < .00005 |  |
| Motivational-volitional patterns (*n* = 1146) | | | | | | | |
| 1 | -8836.21 | 10 | 17742.87 | 1.00 | / | / |  |
| 2 | -8061.17 | 16 | 16235.04 | 0.89 | < .00005 | < .00005 |  |
| 3 | -7807.68 | 22 | 15770.33 | 0.82 | < .00005 | < .00005 |  |
| 4 | -7733.38 | 28 | 15663.99 | 0.76 | .09 | < .00005 |  |
| 5 | -7676.16 | 34 | 15591.81 | 0.80 | .01 | < .00005 |  |
| 6 | -7645.41 | 40 | 15572.57 | 0.81 | .64 | < .00005 |  |
| 7 | -7602.17 | 46 | 15528.37 | 0.81 | .09 | < .00005 |  |
| 8 | -7564.48 | 52 | 15495.26 | 0.83 | .17 | < .00005 |  |

*Note.* LL = Log likelihood; df = number of freely estimated parameters; BIC = Bayens information criterion; VLMR = Vuong-Lo-Mendell-Rubin likelihood ratio test; BLRT = bootstrap likelihood ratio test.

Latent profile analysis was conducted with correction for nesting and the following model specification: class-invariant, diagonal Σ.

^1^The BLRT could only be performed without correction for nesting.
