# Supplementary material for: The interaction of behavioral context and motivational-volitional factors for exercise and sport in adolescence: patterns matter
Source: BMC Public Health. 2020 Apr 28;20:570. doi: 10.1186/s12889-020-08617-5 (PMC7189603; doi:10.1186/s12889-020-08617-5)
Supplement: Supplementary file 2 — Additional file 2 : ESM 2. Split-half-replication of behavioral context and motivational-volitional patterns. [file 12889_2020_8617_MOESM2_ESM.docx]

ESM 2: Split-half-replication of behavioral context and motivational-volitional patterns
