# Supplementary material for: The interaction of behavioral context and motivational-volitional factors for exercise and sport in adolescence: patterns matter
Source: BMC Public Health. 2020 Apr 28;20:570. doi: 10.1186/s12889-020-08617-5 (PMC7189603; doi:10.1186/s12889-020-08617-5)
Supplement: Supplementary file 3 — Additional file 3 : ESM 3. Descriptive statistics of behavioral context and motivational-volitional patterns. [file 12889_2020_8617_MOESM3_ESM.docx]

ESM 3

Table 1

*Descriptive statistics of behavioral context and motivational-volitional patterns on a manifest level*

|  | Behavioral context patterns | |  | |  | |  |
| --- | --- | --- | --- | --- | --- | --- | --- |
| Organizational and social setting factors | P1: Mostly inactive *(n* = 240; 20.94%) | P2: Non-club-organized individualists (*n* = 151; 13.18%) | | P3: Self-organized individualists and family sportpersons (*n* = 254; 22.16%) | P4: Traditional competitive club athletes with friends (*n* = 501; 43.72%) |  | Overall (*N* = 1146) |
|  | *M* (*SD*) | *M* (*SD*) | | *M* (*SD*) | *M* (*SD*) |  | *M* (*SD*) |
| Club-organized | 0.50% (14.10) | 3.70% (14.10) | | 7.50% (14.10) | 87.80% (25.10) |  | 40.60% (14.10) |
| Non-club-organized | 0.60% (10.00) | 85.90% (10.00) | | 1.80% (10.00) | 3.10% (1.80) |  | 13.20% (10.00) |
| Self-organized | 1.00% (13.70) | 9.50% (13.80) | | 88.70% (13.80) | 7.70% (24.40) |  | 24.50% (13.80) |
| Alone | 0.80% (28.10) | 29.50% (28.10) | | 38.20% (28.10) | 9.00% (49.80) |  | 16.50% (28.10) |
| With people you do not know | 1.70% (23.00) | 13.80% (23.00) | | 5.40% (2.30) | 11.60% (40.80) |  | 8.40% (23.00) |
| With family and/or partner | 0.50% (17.00) | 7.60% (17.00) | | 11.10% (1.70) | 3.10% (30.10) |  | 4.90% (17.00) |
| With friends | 9.40% (35.90) | 48.70% (35.90) | | 45.00% (35.90) | 76.20% (63.70) |  | 51.60% (35.90) |
| Competitive participation | 1.40% (31.30) | 12.20% (31.30) | | 13.40% (31.30) | 67.90% (55.40) |  | 34.50% (31.30) |
| Descriptive characteristics of the patterns | | | |  |  |  |  |
| Inactivity | 82.90% | 0.00% | | 0.00% | 0.00% |  |  |
| Age | 15.40 years (*SD* = 0.68) | 15.31 years (*SD* = 0.61) | | 15.32 years (*SD* = 0.70) | 15.21 years (*SD* = 0.60) |  | 15.29 years (*SD* = 0.65) |
| Sex | 60.70% female | 74.10% female | | 51.80% female | 44.60% female |  | 53.40% female |
| Swiss nationality | 72.00% | 79.90% | | 82.10% | 84.90% |  | 80.80% |
| BMI based on self-report | 17.74 (*SD* = 3.16) | 17.66 (*SD* = 2.50) | | 17.50 (*SD* = 2.63) | 17.63 (*SD* = 2.64) |  | 17.63 (*SD* = 2.66) |
|  | Motivational-volitional patterns | | | |  |  |  |
| Motivational-volitional factors | P1: The intention- and plan-less (*n* = 61; 5.32%) | P2: The low motivated with low volition (*n* = 152; 13.26%) | | P3: The moderately motivated with moderate volition (*n* = 414; 36.13%) | P4: The plan-less motivated (*n* = 42; 3.67%) | P5: The highly motivated with high volition (*n* = 477; 41.62%) | Overall (*N* = 1146) |
|  | *M* (*SD*) | *M* (*SD*) | | *M* (*SD*) | *M* (*SD*) | *M* (*SD*) | *M* (*SD*) |
| Self-determined motivation | 2.12 (0.70) | 3.24 (0.70) | | 4.23 (0.70) | 4.59 (0.70) | 5.08 (0.70) | 4.03 (2.53) |
| Non-self-determined motivation | 1.63 (0.97) | 2.27 (0.97) | | 2.34 (0.97) | 2.05 (0.97) | 2.46 (0.97) |  |
| Intention strength | 2.70 (1.14) | 5.90 (1.14) | | 8.05 (1.14) | 8.92 (1.14) | 9.31 (1.14) | 8.02 (2.03) |
| Maintenance self-efficacy | 2.17 (0.58) | 2.60 (0.58) | | 3.07 (0.58) | 3.33 (0.58) | 3.89 (0.58) | 3.30 (0.79) |
| Action planning | 1.25 (0.57) | 2.59 (0.57) | | 3.59 (0.57) | 1.82 (0.57) | 4.36 (0.57) | 3.54 (1.10) |
| Descriptive characteristics of the patterns | | | |  |  |  |  |
| Inactivity | 77.00% | 48.03% | | 14.03% | 21.40% | 2.50% |  |
| Age | 15.42 years (*SD* = 0.77) | 15.37 years (*SD* = 0.74) | | 15.26 years (*SD* = 0.77) | 15.49 (*SD* = 1.10) | 15.24 (*SD* = 0.72) | 15.29 years (*SD* = 0.65) |
| Sex | 43.20% female | 68.60% female | | 56.00% female | 28.90% female | 50.30% female | 53.40% female |
| Swiss nationality | 80.40% | 75.90% | | 80.90% | 92.90% | 81.10% | 80.80% |
| BMI based on self-report | 17.66 (*SD* = 3.52) | 17.51 (*SD* = 3.43) | | 17.52 (*SD* = 3.42) | 17.52 (*SD* = 2.83) | 17.80 (*SD* = 2.95) | 17.63 (*SD* = 2.66) |

*Note.* BMI = body mass index.
