# Supplementary figures and images for: The interaction of behavioral context and motivational-volitional factors for exercise and sport in adolescence: patterns matter
Source: BMC Public Health. 2020 Apr 28;20:570. doi: 10.1186/s12889-020-08617-5 (PMC7189603; doi:10.1186/s12889-020-08617-5)

*
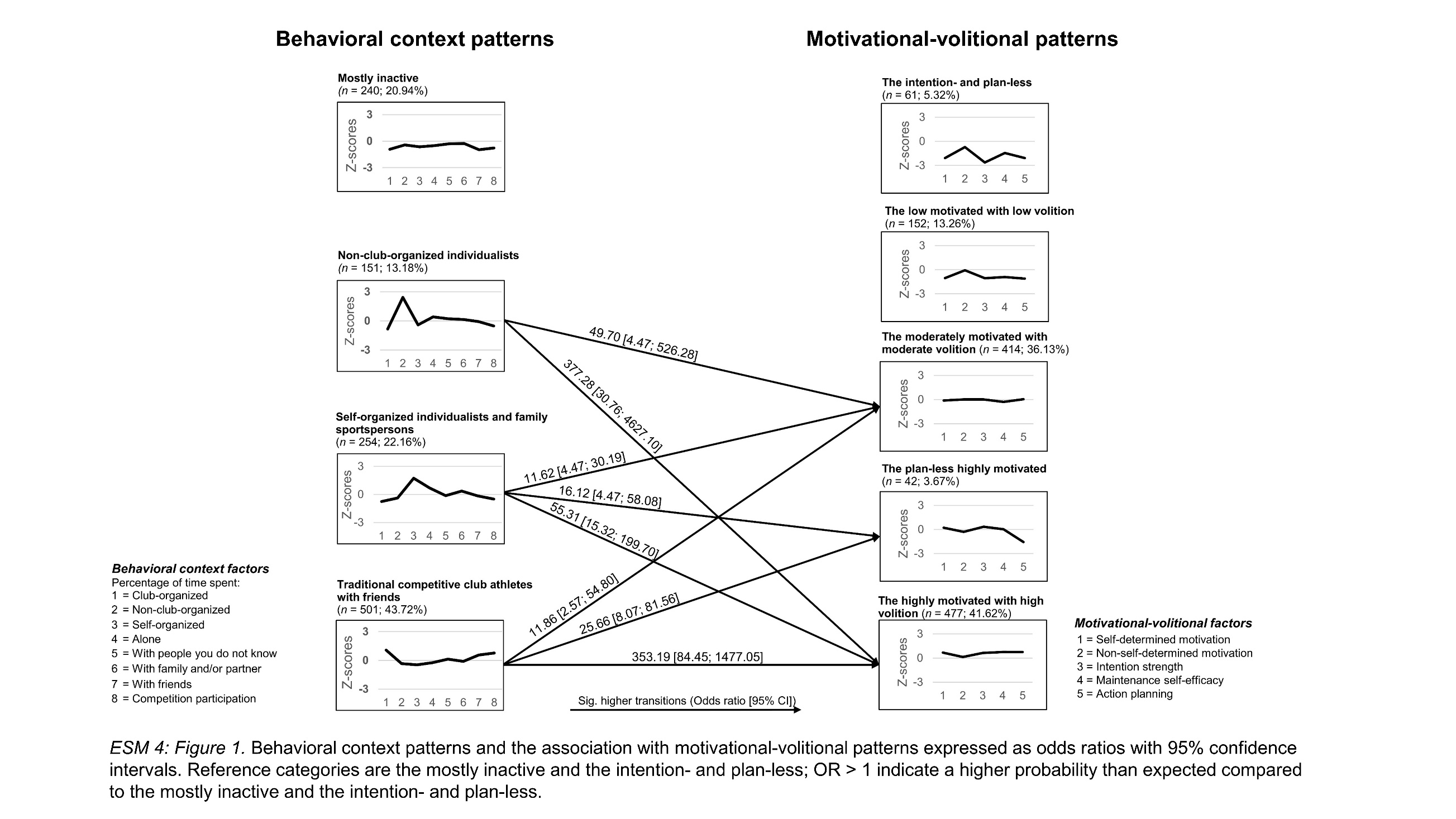
*

Supplement: Supplementary file 4 — Additional file 4 : ESM 4. Behavioral context patterns and the association with motivational-volitional patterns expressed as odds ratios with 95% confidence intervals. [file 12889_2020_8617_MOESM4_ESM.docx]
